# Deconstructing the role of MALAT1 in MAPK-signaling in melanoma: insights from antisense oligonucleotide treatment

**DOI:** 10.18632/oncotarget.28447

**Published:** 2023-05-26

**Authors:** Valentin Feichtenschlager, Yixuan James Zheng, Wilson Ho, Linan Chen, Ciara Callanan, Christopher Chen, Albert Lee, Jose Ortiz, Klemens Rappersberger, Susana Ortiz-Urda

**Affiliations:** ^1^Department of Dermatology, Mt Zion Cancer Research Center, University of California San Francisco, San Francisco, CA 94110, USA; ^2^Department of Dermatology, Clinic Landstrasse Vienna, Academic Teaching Hospital, Medical University Vienna, Vienna, Austria; ^3^School of Medicine, University of California San Francisco, San Francisco, CA 94110, USA

**Keywords:** MALAT1, MAPK-pathway, BRAF, melanoma, antisense oligonucleotides

## Abstract

The long non-coding RNA (lncRNA) MALAT1 is a regulator of oncogenesis and cancer progression. MAPK-pathway upregulation is the main event in the development and progression of human cancer, including melanoma and recent studies have shown that MALAT1 has a significant impact on the regulation of gene and protein expression in the MAPK pathway. However, the role of MALAT1 in regulation of gene and protein expression of the MAPK-pathway kinases RAS, RAF, MEK and ERK in melanoma is largely unknown. We demonstrate the impacts of antisense oligonucleotide (ASO)-based MALAT1-inhibition on MAPK-pathway gene regulation in melanoma. Our results showed that MALAT1-ASO treatment decreased BRAF RNA expression and protein levels, and MALAT1 had increased correlation with MAPK-pathway associated genes in melanoma patient samples compared to healthy skin. Additionally, drug-induced MAPK inhibition upregulated MALAT1-expression, a finding that resonates with a paradigm of MALAT1-expression presented in this work: MALAT1 is downregulated in melanoma and other cancer types in which MALAT1 seems to be associated with MAPK-signaling, while MALAT1-ASO treatment strongly reduced the growth of melanoma cell lines, even in cases of resistance to MEK inhibition. MALAT1-ASO treatment significantly inhibited colony formation *in vitro* and reduced tumor growth in an NRAS-mutant melanoma xenograft mouse model *in vivo*, while showing no aberrant toxic side effects. Our findings demonstrate new insights into MALAT1-mediated MAPK-pathway gene regulation and a paradigm of MALAT1 expression in MAPK-signaling-dependent cancer types. MALAT1 maintains essential oncogenic functions, despite being downregulated.

## INTRODUCTION

Melanoma is the deadliest form of skin cancer with a steadily increasing incidence rate. Approximately 2.1 percent of men and women will be diagnosed with melanoma of the skin during their lifetime [1]. Despite recent advancements in melanoma therapy, such as small molecule inhibitors that target the MAPK-pathway as well as PD1-inhibitors, the 5-year survival rate for distant metastasized melanoma is only 31.9% [1–3]. The MAPK-pathway is a critical signaling pathway in human cells that regulates cell proliferation, survival, and death [4]. In normal cells this pathway is tightly regulated, but many human tumors have mutations that disrupt MAPK-signaling, leading to increased cell growth, invasion, metastasis, and angiogenesis [4]. In melanomas, this dysregulation is often caused by activating mutations in the BRAF and NRAS genes [4].

While most of the human genome is transcribed, a large segment of the transcriptome does not obtain protein-coding functions [5, 6]. Long non-coding RNAs (lncRNAs) are a class of non-coding genomic sequences that are larger than 200 nucleotides [6]. Historically, non-coding RNA was thought to be non-functional “junk” RNA [6]. However, recent research has revealed that lncRNAs play important roles in both normal and cancer-related cellular processes [7, 8]. MALAT1 (Metastasis Associated Lung Adenocarcinoma Transcript 1), also known as NEAT2 (Non-coding Nuclear-enriched Abundant Transcript 2), is an oncogenic lncRNA that was first identified in non-small cell lung cancer, and has since been found to play essential roles in progression, metastasis, and survival of various types of cancer, including melanoma [9–15]. It has been shown that MALAT1 contributes to various physiological and pathological processes as a regulator of the MAPK-pathway. For example, MALAT1-inhibition resulted in conspicuous inhibition of MAPK-signaling and MALAT1 activates MAPK-signaling during the early phase of neuronal differentiation [16]. MALAT1 plays a role in UVB-induced photo-aging via MAPK-signaling regulation [17]. It also regulates proliferation and cell cycle through MAPK-signaling in endometriosis granulosa cells [18]. Furthermore, inhibition of MALAT1 after myocardial infarction has been shown to significantly improve the cardiac function of rats by downregulating the MAPK-pathway [19]. In gallbladder cancer and hepatocellular carcinoma, it has been reported that MALAT1 plays an oncogenic role as a promoter of proliferation and metastasis through activating the MAPK-pathway [14, 15]. Additionally, increased MALAT1 expression has also been found to promote the proliferation, migration, and invasion of non-small cell lung cancer via the MAPK-signaling pathway [20]. However, in contrast, MALAT1 may have a tumor-suppressive function in glioma cells through inactivation of MAPK-signaling [21]. Together, these findings suggest that the MAPK-signaling/MALAT1 axis has essential and tissue-specific oncogenic roles in cancer.

Synthetic oligonucleotides such as Antisense Oligonucleotides (ASOs) and small interfering RNAs (siRNAs) are commonly used to reduce lncRNA levels [22]. While siRNA-mediated degradation of target RNA is thought to happen mainly in the cytosolic compartment of the cell, ASOs can degrade RNA in the cytoplasm and in the nucleus [23–25]. ASOs are single-stranded DNA structures that bind to their target RNA and the formation of this hetero-duplex structure activates RNase H-mediated RNA degradation, allowing for specific reduction of RNA levels [25]. ASOs can be modified chemically to reduce toxicity, increase stability and increase target affinity, which has led to multiple ongoing clinical trials and successful U.S. Food and Drug Administration (FDA) approvals for ASO-based therapies [26–28]. There have been several promising preclinical studies using ASOs and siRNAs to target MALAT1 in various human tumors, including melanoma [11, 29–39].

In this study, we present novel transcriptional dependencies between MALAT1 and MAPK-pathway-associated genes in melanoma. We demonstrate the specificity of an ASO-based approach to downregulate MALAT1, which leads to downregulation of BRAF gene and protein expression. Additionally, we show that drug-induced inhibition of MAPK-signaling leads to significant and dose-dependent upregulation of MALAT1. Using a large panel of patient-derived tissue samples, we further show that MALAT1 expression correlates significantly with RNA expression of genes coding for the MAPK-pathway key kinases NRAS, BRAF, MEK1/2 and ERK1/2 in healthy skin, and notably to a greater extent in melanoma. Furthermore, we present a paradigm in MALAT1 expression: MALAT1 is reported to regulate MAPK-signaling and promote oncogenic functions in melanoma, liver, and lung cancer. We show that MALAT1 is strongly downregulated in these cancer types. However, using a large panel of NRAS- and BRAF-mutated cell lines as a model for MAPK-dependent melanoma, we show that MALAT1 expression is essential for melanoma colony formation, cell growth, and tumor growth. These findings are consistent with our results that show MALAT1 upregulation upon drug induced MAPK-signaling inhibition. Acquired treatment resistance to MEK-inhibition does not affect MALAT1 expression and resistant cell lines remain vulnerable to MALAT1 inhibition. In summary, the presented data provide new insights into the regulatory function of MALAT1 on MAPK-pathway associated gene expression in melanoma and highlights that MALAT1 maintains essential oncogenic functions while simultaneously being downregulated.

## RESULTS

### ASO-mediated targeting of MALAT1 has high “on-target” and low “off-target” specificity.

**Figure 1:**
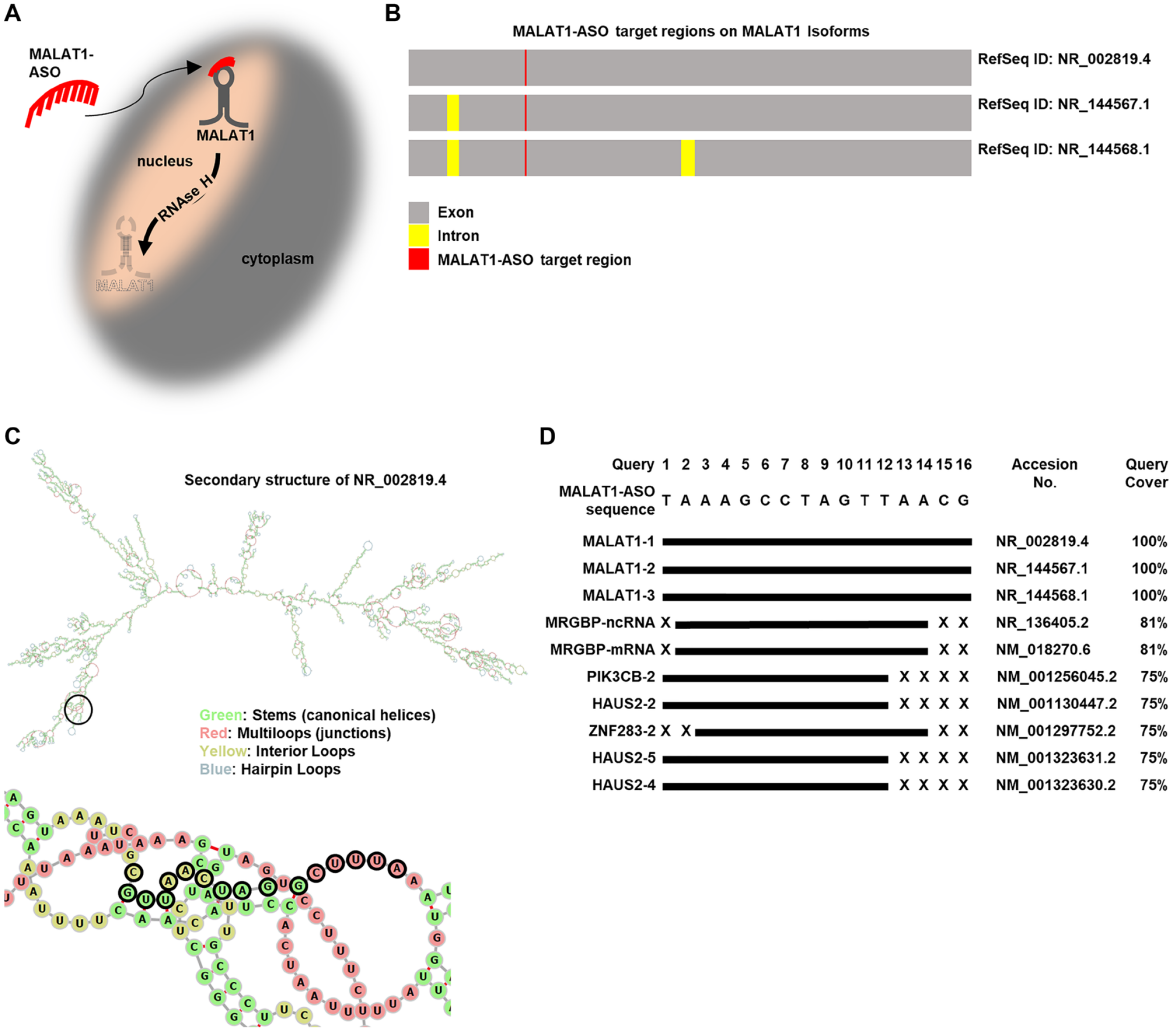
MALAT1-ASO specifically targets MALAT1-lncRNAs. (**A**) Schematic illustration of the MALAT1-ASO bypassing the cell-membrane and entering the nucleus of melanoma cells to inhibit MALAT1 through target-binding and RNAse H mediated degradation. (**B**) Schematic illustration of the three MALAT1 isoforms that are targeted by MALAT1-ASO, highlighting intron, exon and MALAT1-ASO target-binding regions. (**C**) Top: Secondary RNA-structure (MFE) of NR_002819.4 and the target binding region of the MALAT1-ASO (black circle). Below: Selected cutout and zoom of the secondary structure of the target region, showing that the MALAT1-ASO target site is an accessible structure for RNA-ASO binding. The nucleotides that are targeted by the MALAT1-ASO sequence are highlighted with black outline. Contrary to the complete structure (top), the selected cutout (bottom) does not include hairpin loops. (**D**) The top10 hits of matching targets to the MALAT1-ASO sequence in the human transcriptome show high specificity of MALAT1-ASO to MALAT1 isoforms. Other targets have at least three mismatches, indicating a low “off-target” binding probability of the MALAT1-ASO. Targets are ranked by the expect value (E-value). The graphical illustration of the target sequences corresponds to their mRNA sequences.

Our aim was to study the regulatory functions of MALAT1 on gene expression of MAPK-pathway key kinases in melanoma. Previous studies have shown that MALAT1 is highly enriched in the nuclei of human cells, including NRAS mutant melanoma [40, 41]. To reduce MALAT1 RNA levels, we used an ASO-based approach, which is a laboratory method that has the potential to be translated into clinical therapeutic approaches. ASOs are singe-stranded complexes that bind to their target RNA, resulting in the recruitment of RNase H and RNA degradation [25]. A MALAT1-targeting GapmeR antisense oligonucleotide was constructed and Figure 1A provides a schematic illustration of the nuclear-localized lncRNA MALAT1 and its RNase H-dependent degradation using a MALAT1-targeting ASO of the GapmeR type. Following the Ensembl gene annotation GRCH38.p13 (human) and the LINCPEDIA-dataset for long non-coding RNAs (V 5.2), we found low coding potential for all 17 different isoforms of MALAT1 listed in the Ensembl database. (Supplementary Table 1) The MALAT1-ASO targets three different MALAT1 isoforms (NR_002819.4, NR_144567.1, and NR_144568.1), as visually displayed in Figure 1B. The Ensembl canonical transcript ENST00000534336 (Accession Nr.: NR_002819.4) is the most conserved and highest expressed representative MALAT1-transcript, consisting of 1 exon with a length of 8762 bp. We chose this isoform, to visually present the target region of the MALAT1-ASO on a computational generated secondary structure construct of MALAT1. (Figure 1B, 1C, Supplementary Table 1). Due to the thermodynamic stability of an RNA structure, the local regions that allow the formation of a stable ASO-RNA hybrid are usually located at structures such as interior loops, joint sequences, hairpins and bulges of 10 or more consecutive nucleotides [42]. The MALAT1-ASO targets a region that contains of accessible structures including stem-, multiloop- and interior loop-regions. (Figure 1C). Next, we aimed to analyze the potential “off-target” toxicity caused by the binding of the MALAT1-ASO to RNAs other than MALAT1. Previous studies have shown that that the efficacy of GapmeR-ASOs, as used in our study, is strongly reduced after one mismatch, and two mismatches lead to the complete inactivation of the ASOs [43]. Screening the MALAT1-targeting ASO sequence against the human transcriptome shows that the three MALAT1-isoforms are targeted by the MALAT1-ASO sequence with 100 % specificity. Importantly, the MALAT1-ASO sequence does not show any “off-target hits” with one or two mismatches. The MALAT1-ASO sequence only shows “off-target” hits with at least three mismatches in their sequence. (Figure 1D, Supplementary Table 2) Together, these results indicate a high potential for the presented MALAT1-ASO sequence to cause a profound inhibition of MALAT1, while simultaneously showing a low likelihood of causing “off-target” toxicity.

### MALAT1-ASO treatment downregulates MALAT1 and BRAF RNA levels in melanoma, while MAPK-downregulation increased MALAT1-expression

**Figure 2:**
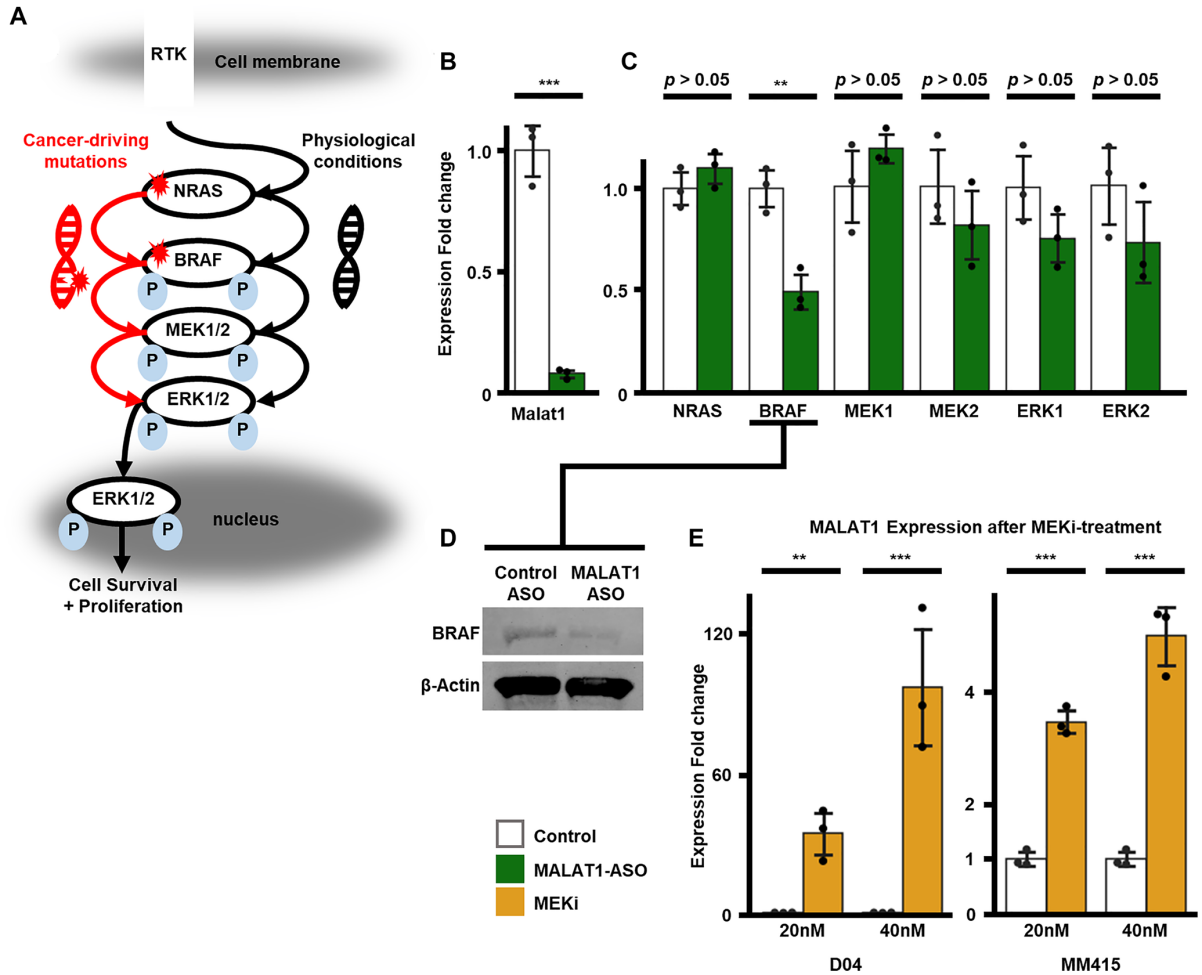
Relative fold enrichment analysis of MAPK-pathway genes and MALAT1. (**A**) A schematic illustration of activation and phosphorylation cascade of the RAS/RAF/MEK/ERK (MAPK) signaling pathway. Under physiological conditions, a receptor tyrosine kinase (RTK) which is located in the cellular membrane, activates NRAS. NRAS activates BRAF through phosphorylation (P), which activates MEK1/2, which then activates ERK1/2. Activated ERK1/2 phosphorylates downstream targets which regulate cell survival, proliferation and apoptosis and transfers into the nucleus. Alternatively, activating mutations in NRAS and BRAF can auto-activate the proteins and hyperactivate the MAPK-pathway, without the requirement of ligand mediated activation. (**B**) Strong (11-fold, SD = 0.02) and significant (*p* < 0.001) downregulation of MALAT1 RNA levels was measured in D04 cells treated with non-targeting control ASO versus D04 samples treated with MALAT1-ASO. Final ASO concentration in media was 50 nM for a treatment period of 24 hours. (**C**) When comparing D04 samples treated with non-targeting control ASO versus D04 samples treated with MALAT1-ASO, significant RNA-expression downregulation of the MAPK-signaling kinase BRAF (1.01-fold decrease, SD = 0.01, *p* = 0.006) was measured, while no significant expression alteration was measured for NRAS (0.1-fold increase, SD = 0.09, *p* = 0.13), MEK1 (0.2-fold increase, SD = 0.09, *p* = 0.14), MEK2 (0.23-fold decrease, SD = 0.2, *p* = 0.16), ERK1 (0.33-fold decrease, SD = 0.14, *p* = 0.07) and ERK2 (0.37-fold decrease, SD = 0.24, *p* = 0.11). Final ASO concentration in media was 50 nM for a treatment period of 24 hours. (**D**) Immunoblotting showing a decrease of BRAF protein levels 24 hours after MALAT1-ASO treatment compared to Control-ASO treatment in D04 cell lysate (50 nM final ASO concentration). β-actin served as a loading control. (**E**) MEKi caused significant and dose dependent MALAT1-upregulation. D04 cells responded with 34-fold upregulation (SD = 10.87, *p* = 0.001) to 20 nM MEKi treatment and 96-fold enrichment (SD = 30.49, *p* < 0.001) to 40 nM MEKi treatment. MM415 cells are less vulnerable to MEKi treatment and reacted with 2.5-fold increase (20 nM; SD = 0.25, *p* < 0.001), respectively 4-fold increase (40 nM; SD = 0.63, *p* < 0.001) of MALAT1-expression. Cells were either treated with trametinib (MEKi) or DMSO (control). Treatment period was 72 hours. For (B, C, E) CT-values were normalized to GAPDH, and fold enrichment was calculated using the 2^–ΔΔCt^ method. Error bars represent standard deviation (SD). All experiments were performed in triplicates (*n* = 3/group). Significance is shown as *p*-values calculated by Students *t*-test. ^*^ *p* < 0.05, ^**^ *p* < 0.01, ^***^ *p* < 0.001.

Figure 2A presents a simplified diagrammatic representation of the molecular activation steps and interactions in the RAS/RAF/MEK/ERK (MAPK) kinase cascade. Ligand-induced stimulation of a receptor tyrosine kinase (RTK) situated on the plasma membrane activates RAS. Subsequently, RAS activates RAF through phosphorylation. RAF, in turn, phosphorylates MEK1/2, leading to their activation. MEK1/2 activation results in phosphorylation and activation of ERK1/2. Once activated, ERK1/2 phosphorylates various downstream targets localized in different subcellular compartments, ultimately regulating crucial cellular processes such as cell survival, proliferation, and apoptosis [44]. In melanoma and other types of cancer, activating mutations in NRAS and BRAF can constitutively auto-activate the kinases without the requirement of ligand mediated activation, causing hyperactivation of the pathway [44]. To confirm the effectiveness of the MALAT1-targeting ASO in MALAT1-inhibition, we exposed NRAS-mutant D04 melanoma cells to non-targeting Control-ASO or MALAT1-ASO at a concentration of 50 nM for one day. We then extracted RNA and performed RT-qPCR. Our results showed a significant 11-fold decrease in MALAT1 RNA levels (*p* < 0.001, SD = 0.02, Figure 2B). Next, we aimed to investigate the impact of MALAT1 inhibition on genes that code for key kinases in the MAPK pathway. Using RT-qPCR we measured changes in RNA levels of NRAS, BRAF, MEK1, MEK2, ERK1 and ERK2, caused by MALAT1-ASO treatment compared to treatment with the Control-ASO with an equal concentration and incubation time of 50 nM and 1 day. As a result of MALAT1-ASO treatment, there were no significant changes in the RNA levels of NRAS (0.1-fold increase, SD = 0.09, *p* = 0.13), MEK1 (0.2-fold increase, SD = 0.09, *p* = 0.14), MEK2 (0.23-fold decrease, SD = 0.2, *p* = 0.16), ERK1 (0.33-fold decrease, SD = 0.14, *p* = 0.07) and ERK2 (0.37-fold decrease, SD = 0.24, *p* = 0.11). However, there was a significant reduction of RNA levels of BRAF. (1.01-fold decrease, SD = 0.1, *p* = 0.006, Figure 2C) To determine if this effect also impacted BRAF protein expression, we examined protein levels of BRAF one day after MALAT1-ASO treatment at a final concentration of 50 nM. Using Immunoblot, it was found that BRAF protein was downregulated upon MALAT1-inhibition (Figure 2D).

To investigate additional co-regulatory mechanisms of MALAT1 expression and MAPK signaling, we measured the response of MALAT1 expression to drug-induced MAPK inhibition in two NRAS-mutant melanoma cell lines, D04 and MM415. Trametinib, a MEK1 and MEK2 kinase inhibitor (MEKi), was used to downregulate MAPK signaling by treating cells with 20 nM or 40 nM of the drug for 3 days. Previously published data established that trametinib significantly suppresses MAPK signaling in both cell lines, as evidenced by strong reduction phospho-ERK levels upon treatment [45]. Trametinib is an allosteric, non-ATP-competitive inhibitor of the MEK1 and MEK2 kinases, and it was the first MEKi that received FDA approval for the treatment of melanoma. Additionally, it was later approved for use in combination with BRAF inhibitors and has become a mainstay for the treatment of patients with unresectable or metastatic melanoma with BRAF mutations [46]. In the D04 cell line, treatment with 20 nM trametinib led to a significant 34-fold increase (*p* = 0.001, SD = 10.87) of MALAT1 expression. A treatment of 40 nM over the same time period resulted in an even greater increase of 96-fold. (*p* < 0.001, SD = 30.49, Figure 2E) The MM415 cell line is less sensitive to MEKi treatment compared to D04 [45]. Therefore, the increase in MALAT1 expression after trametinib treatment was weaker compared to D04. However, the MM415 cell line responded similarly, with dose-dependent and significant 2.5-fold (20 nM: *p* < 0.001, SD = 0.25) or 4-fold (40 nM: *p* < 0.001, SD = 0.63) increase of MALAT1 expression (Figure 2E).

Efficient inhibition of MALAT1 is crucial for investigating its role in gene regulation. Our results demonstrate that the MALAT1-ASO sequence we used effectively reduces MALAT1 RNA levels *in vitro*. We also observed a decrease in BRAF RNA levels and protein expression upon MALAT1 inhibition in melanoma. Furthermore, our findings indicate that MALAT1 expression is strongly and dose-dependently upregulated in response to MAPK pathway signaling inhibition.

### Gene-expression of MALAT1 and MAPK-pathway kinases correlates in patient derived healthy skin samples and to a greater extent in melanoma

**Figure 3:**
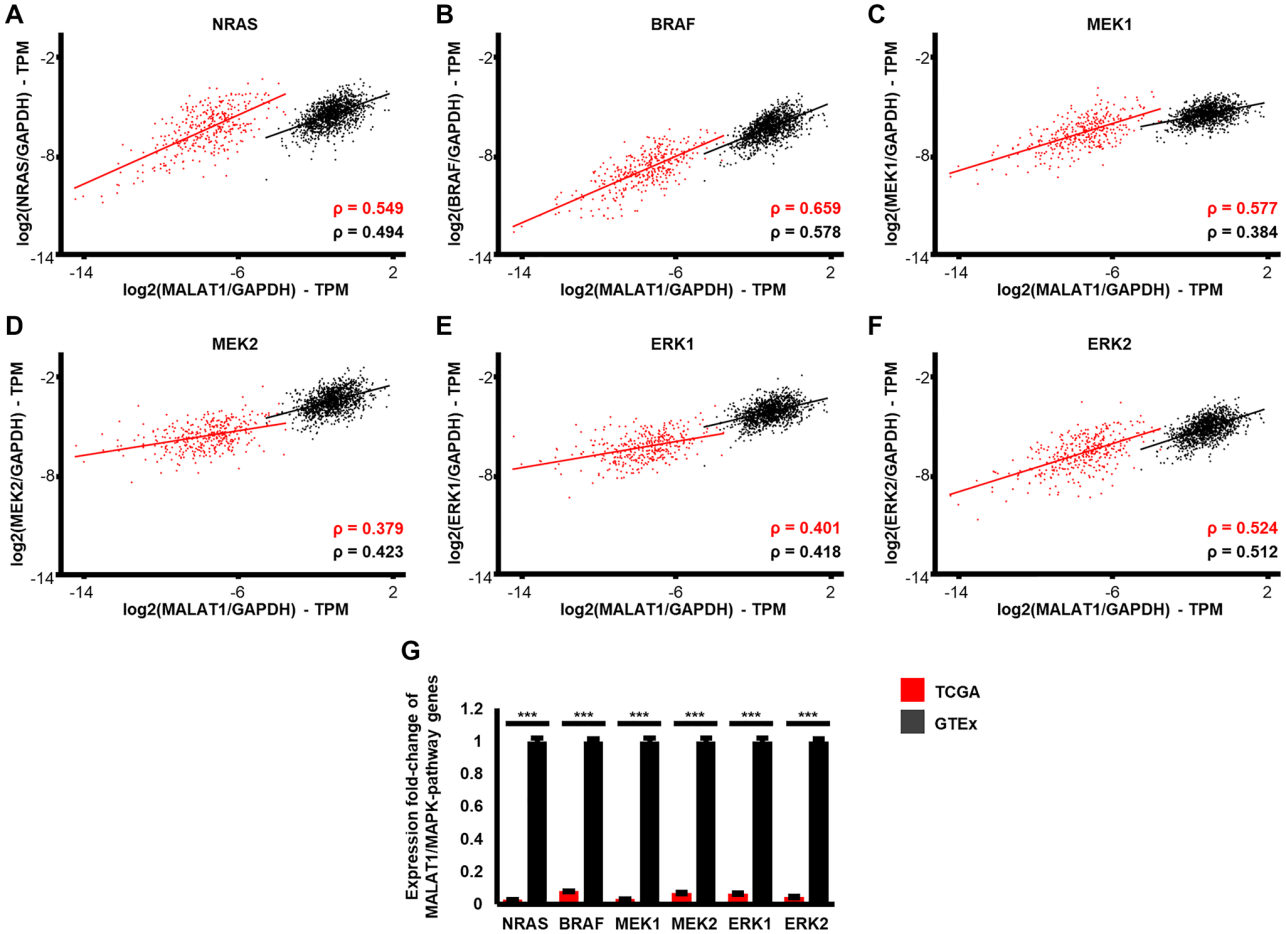
Expression patterns of MALAT1 and MAPK-signaling associated genes in patient derived healthy skin compared to NRAS and BRAF mutated melanoma samples. Expression-correlation is shown for MALAT1 and the proto-oncogenes (**A**) NRAS, (**B**) BRAF, (**C**) MEK1, (**D**) MEK2, (**E**) ERK1, and (**F**) ERK2. The black (GTEx database, *n* = 1305) and red (TCGA database, *n* = 366) lines represent Spearman correlation (ρ). Expression correlation was significant (*p* < 0.001) in all comparisons. Expression correlation was stronger in melanoma than healthy skin in all comparisons, except MALAT1-MEK2 and MALAT1-ERK1. Expression values of genes in (A–F) were normalized to GAPDH. (**G**) MALAT1 is significantly reduced in melanoma samples compared to healthy skin, when put into relation to expression to the MAPK-pathway genes NRAS (38-fold, *p* < 0.001), BRAF (13-fold, *p* < 0.001), MEK1 (33-fold, *p* < 0.001), MEK2 (15-fold, *p* < 0.001), ERK1 (16-fold, *p* < 0.001) and ERK2 (23-fold, *p* < 0.001). Error bars represent standard error of the mean and significance is shown as *p*-values calculated by Students *t*-test. ^*^ *p* < 0.05, ^**^ *p* < 0.01, ^***^ *p* < 0.001.

To gain further insight into the potential interactions between MALAT1 and MAPK pathway signaling regulators, we analyzed the expression patterns of MALAT1 with NRAS, BRAF, MEK1, MEK2, ERK1 and ERK2 in a large dataset of healthy skin and melanoma patient samples. We used The Genotype-Tissue Expression (GTEx) database (*n* = 1305) for the analysis of non-cancerous patient skin samples and The Cancer Genome Atlas (TCGA) database (*n* = 366) for the analysis of NRAS- and BRAF-mutated melanoma patient samples. We used Spearman rank correlation (ρ) to measure the association between gene expressions. Our results demonstrate that NRAS expression is significantly correlated with MALAT1 expression in both non-cancerous skin (*p* < 0.001) and melanoma (*p* < 0.001). The correlation between MALAT1 and NRAS expression was high in both comparisons, and even higher in melanoma. (ρ = 0.494 vs. 0.549, Figure 3A) This is significant because NRAS mutations are present in 15–30% of melanoma cases and have been linked to drug resistance mechanisms [4, 47, 48]. Furthermore, it was shown, that MALAT1 may act as master regulator of several protein-coding hub-genes in drug-resistant NRAS-mutant cancer [49]. NRAS activates several downstream pathways in the MAPK signaling pathway, such as BRAF, MEK1, MEK2, ERK1 and ERK2. (Figure 2A) Approximately 50–70% of melanoma patients harbor cancer driving mutations in the BRAF gene, making it a powerful therapeutic target in melanoma treatment [4, 48]. When BRAF-signaling is activated in melanoma, it downregulates the Wnt/β-catenin pathway, leading to inhibition of apoptosis in melanoma cells [50]. Studies have shown that MALAT1 may play a similar role in colon cancer cells by inhibiting Wnt/β-catenin signaling [51]. Correlation analysis between MALAT1 and BRAF expression identified similar patterns to MALAT1 and NRAS expression. MALAT1 and BRAF expression was significantly correlated in both non-cancerous skin (*p* < 0.001) and in melanoma (*p* < 0.001), with the correlation being even stronger in melanoma. (ρ = 0.578 vs. 0.659, Figure 3B) MEK and ERK kinases are downstream targets of BRAF in the MAPK signaling pathway. (Figure 2A) We found that MALAT1 and MEK1 expression were significantly correlated in both healthy skin samples and melanoma (both *p* < 0.001). The correlation was again stronger in melanoma (ρ = 0.577) compared to healthy skin. (ρ = 0.384; Figure 3C) MALAT1 and MEK2 expression also showed a significant correlation in both healthy skin and melanoma (both *p* < 0.001). Interestingly, this correlation was slightly stronger in healthy skin (ρ = 0.423) compared to melanoma. (ρ = 0.379; Figure 3D) Previous studies have shown that MALAT1 serves as a regulator of activation of ERK1/2 in photo-aged fibroblasts and hepatic cellular carcinoma [15, 17]. Here, we found that MALAT1 expression was significantly correlated with ERK1 and ERK2 expression in both healthy skin and melanoma (all four comparisons: *p* < 0.001). The correlation between MALAT1 and ERK1 was similar in strength in both melanoma (ρ = 0.401) and healthy skin. (ρ = 0.418; Figure 3E) MALAT1 and ERK2 expression also showed a strong correlation in both healthy skin (ρ = 0.512) and melanoma. (ρ = 0.524; Figure 3F) In a next step we analyzed the TCGA (melanoma) and GTEx (healthy skin) databases for potential alterations of gene expression of MALAT1 in relation to NRAS, BRAF, MEK1, MEK2, ERK1 and ERK2 gene expression. The relative expression of MALAT1 to all genes was significantly and strongly decreased in melanoma, when compared to healthy skin (NRAS: 38-fold; BRAF: 13-fold, MEK1: 33-fold, MEK2: 15-fold, ERK1: 16-fold, ERK2: 23-fold, for all comparisons: *p* < 0.001, Figure 3G).

The MAPK-pathway is a critical signaling pathway in cancer that is tightly regulated in non-cancerous cells [4]. The large-scale analysis presented here indicates that the correlation of MALAT1 and MAPK kinase gene expression increases in melanoma and the relative expression of MALAT1 to the MAPK-pathway genes NRAS, BRAF, MEK1, MEK2, ERK1 and ERK2 is strongly reduced in melanoma, when compared to healthy skin.

### The paradigm of MALAT1-expression in melanoma and other types of cancer

**Figure 4:**
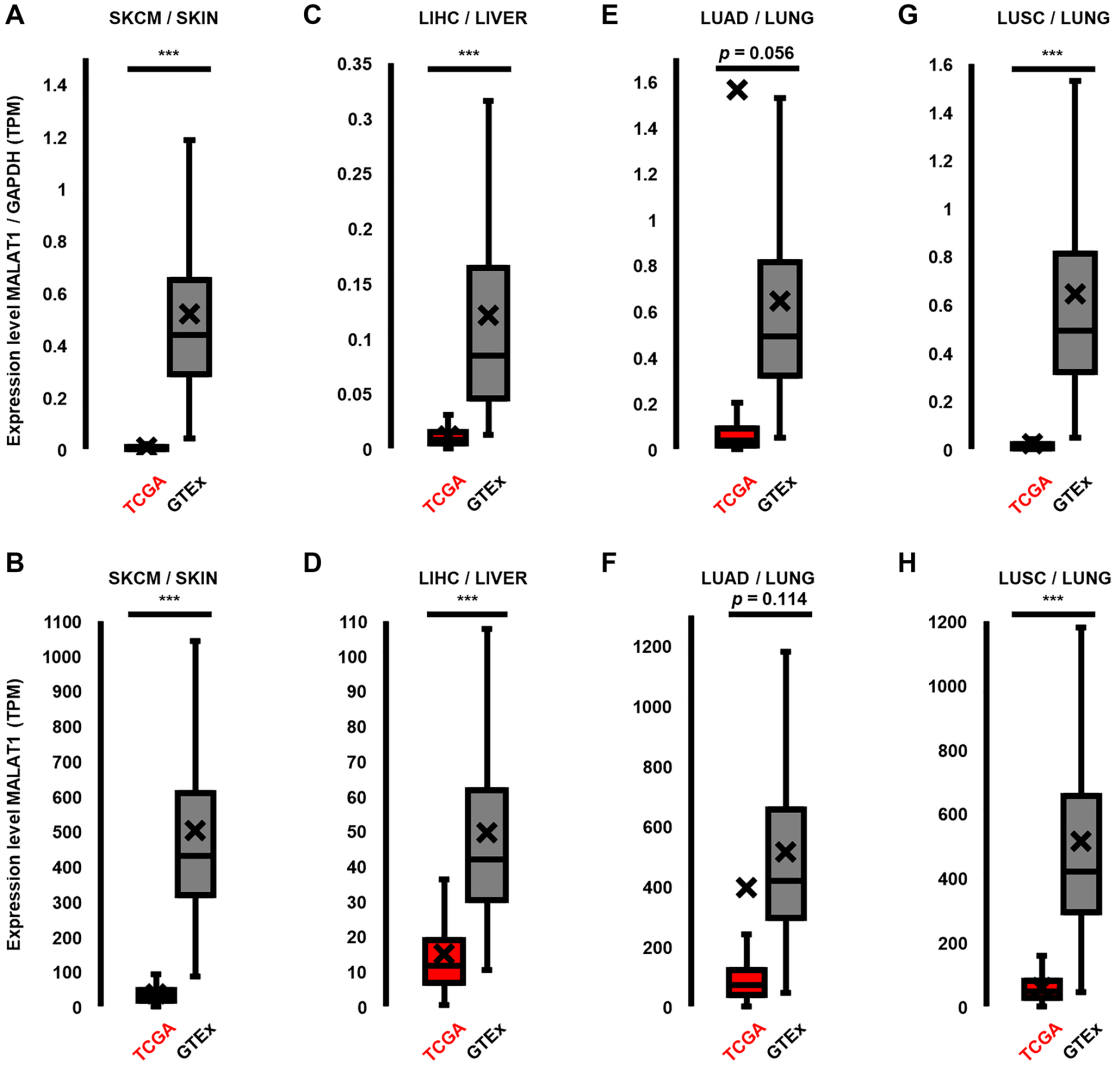
MALAT1 is downregulated in cancer types in which it is associated with the MAPK pathway. (**A**, **B**) In patient-derived tissue samples of NRAS and BRAF mutated melanoma, MALAT1 expression was significantly and strongly reduced by 63-fold (normalized)/14-fold (not normalized, both: *p* < 0.001), compared to healthy skin. (**C**, **D**) Similarly, in liver hepatocellular carcinoma (LIHC), MALAT1 expression was significantly reduced by 9.2-fold (normalized)/3-fold (not normalized, both: *p* < 0.001) in comparison to healthy liver tissue. (**E**, **F**) MALAT1 expression in lung adenocarcinoma (LUAD) did not show a significant change when compared to healthy lung tissue (normalized: 1.42-fold increase, *p* = 0.056; not normalized: 0.3-fold decrease, *p* = 0.0114). (**G**, **H**) In lung squamous cell carcinoma (LUSC) MALAT1 expression was significantly reduced by 28-fold (normalized)/9-fold (not normalized, both: *p* < 0.001) in comparison to healthy lung tissue. Center line is median, box spans first quartile (Q1) to third quartile (Q3), whiskers extend to furthest value < 1.5 x IQR from lower/upper quartile. Mean is marked as X. TPM Expression values of genes were normalized to TPM of GAPDH. Significance is shown as *p*-values calculated by Students *t*-test. ^*^ *p* < 0.05, ^**^ *p* < 0.01, ^***^ *p* < 0.001.

Next, we analyzed MALAT1 expression normalized to GAPDH expression by comparing expression levels in the TCGA and GTEx databases. Contrary to our expectations, MALAT1 expression was found to be strongly decreased in melanoma. (MALAT1/GAPDH TPM in GTEx: 0.52 vs. TCGA: 0.01, Figure 4A) Specifically, MALAT1 showed a significant 63-fold decrease in patient-derived NRAS- and BRAF-mutated melanoma (*p* < 0.001, Figure 4A) These findings are particularly noteworthy as previous analyses in smaller sample sizes of melanoma and adjacent normal tissues showed significant overexpression of MALAT1 in melanoma [12, 52]. However, a direct comparison of these divergent findings is not possible, as the mutational backgrounds of the samples in previous studies were not reported.

We aimed to investigate whether this anomaly of MALAT1 downregulation is specific to melanoma. We hypothesized that MALAT1 could also be downregulated in other cancer types that have a regulatory axis in MALAT1/MAPK-signaling. To test this, we expanded the expression analysis of patient-derived tissue in other TCGA-listed types of cancer in which MALAT1 is known to have essential MAPK-signaling regulating functions [15, 20]. We investigated normalized MALAT1 expression in hepatocellular carcinoma and non-small cell lung cancer in tumor (TCGA) and associated healthy tissue biopsies (GTEx). We found that MALAT1 expression was significantly downregulated in liver hepatocellular carcinoma (LIHC, *p* < 0.001, 9.2-fold, Figure 4B), when compared to healthy liver tissue. The two main subtypes of non-small cell lung cancer are lung adenocarcinoma (LUAD) and lung squamous cell carcinoma (LUSC). In LUAD mean MALAT1 expression was increased (1.42-fold), although not significantly (*p* = 0.056, Figure 4C). However, strong (27.8-fold) and significant (*p* < 0.001) decrease of MALAT1 expression was seen in LUSC, when compared to healthy lung tissue. (Figure 4D) A more detailed overview of normalized MALAT1-expression values in each dataset is listed in Table 1. While comparing the expression levels of genes between two different datasets (TCGA and GTEx) the data was normalized to GAPDH to adjust for any technical variations between the datasets. However, extremely high values were found in the 4th quartile of the LUAD-TCGA dataset, which were to a certain degree due to normalization. To provide a clearer picture of the distribution of MALAT1 gene expression levels and alteration of average expression levels through outliers, non-normalized data is presented in Figure 4E–4H and Table 1. As already seen in the normalized data, MALAT1 expression was significantly decreased in BRAF- and NRAS-mutated melanoma (13.5-fold, *p* < 0.001, Figure 4E), LIHC (3.3-fold, *p* < 0.001, Figure 4F) and LUSC (8.7-fold, *p* < 0.001, Figure 4H), when compared to their corresponding healthy tissue. In the non-normalized data comparison MALAT1 is also decreased in LUAD (1.3-fold, Figure 4G); however, the difference in expression levels again is not significant (*p* = 0.11). These data suggest that MALAT1 follows a pattern of decreased expression in cancer types where it plays a crucial role in oncologic functions and interacts with and regulates MAPK pathway signaling.

**Table 1:** Average Expression, Median (Q2, second quartile), standard error of the mean (SE), first quartile (Q1), third quartile (Q3), and fourth quartile (Q4) of MALAT1 expression along with sample number (*N*), in RNA-seq datasets of patient-derived samples

Cancer type	Average	Median (Q2)	SE	Q1	Q3	Q4	*N*
**Cancer (TCGA), MALAT1 TPM, not normalized**							
NRAS-, and BRAF-mutant SKCM	37.17	31.07	1.62	16.64	47.69	293.59	366
LIHC	14.95	11.59	0.62	6.64	18.87	111.35	424
LUAD	396.24	71.68	97.29	39.81	122.01	32898.29	585
LUSC	59.38	48.69	2.12	28.14	80.24	713.98	550
**Cancer (TCGA), MALAT1/GAPDH TPM, normalized**							
NRAS-, and BRAF-mutant SKCM	0.01	0.01	0.00	0.00	0.01	0.08	366
LIHC	0.01	0.00	0.00	0.00	0.02	0.08	424
LUAD	1.56	0.04	0.58	0.02	0.09	259.15	585
LUSC	0.02	0.01	0.00	0.01	0.02	0.39	550
**Healthy Tissue (GTEx), MALAT1 TPM, not normalized**							
Skin	501.71	429.60	7.84	317.60	608.00	2875.00	1305
Liver	49.45	41.95	1.82	30.34	61.44	199.20	226
Lung	514.51	419.50	12.38	295.45	653.90	1679.00	578
**Healthy Tissue (GTEx), MALAT1/GAPDH TPM, normalized**							
Skin	0.52	0.44	0.01	0.29	0.65	3.39	1305
Liver	0.12	0.08	0.00	0.05	0.16	0.78	226
Lung	0.64	0.49	0.02	0.32	0.81	2.83	578

The values are in transcripts per million (TPM). The values listed in this table cover the TCGA datasets of NRAS- and BRAF-mutated melanoma (SKCM), liver hepatocellular carcinoma (LIHC), lung adenocarcinoma (LUAD) and lung squamous cell carcinomas (LUSC) as well as the GTEx datasets for healthy tissue biopsies of skin, liver, and lung. MALAT1 expression values are shown both normalized to GAPDH-expression and not normalized.

### MALAT1-ASO treatment significantly reduces cell growth in melanoma

**Figure 5:**
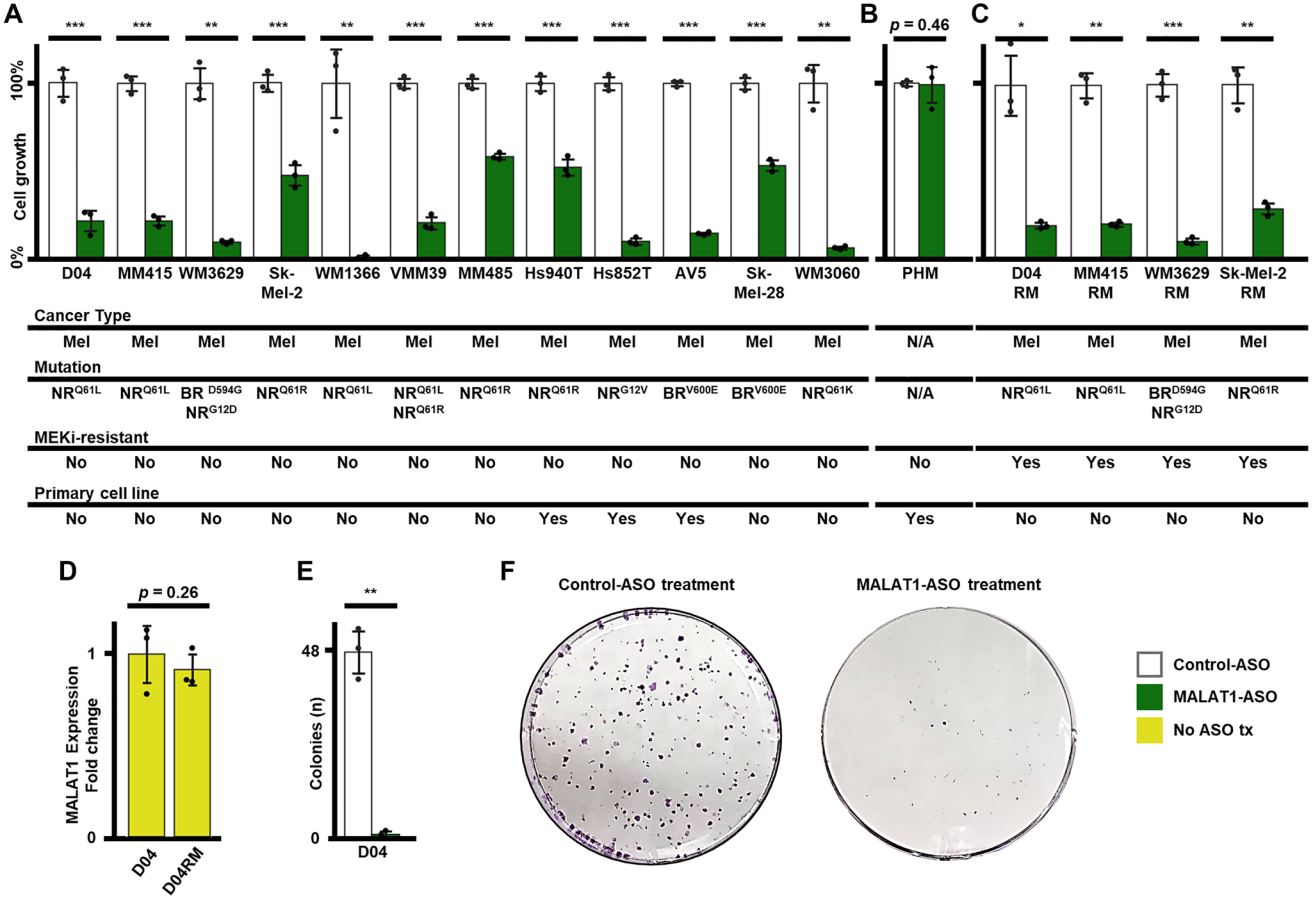
MALAT1-ASO treatment significantly reduces cell-growth in NRAS (NR) and BRAF (BR) mutated melanoma (mel) cells *in vitro*. (**A**) The treatment causes reduction (re) of cell growth in the D04 (re = 77.74%, SD = 7%, *p* < 0.001), MM415 (re = 77.83%, SD = 2.8%, *p* < 0.001), WM3629 (re = 89.83%, SD = 1.4%, *p* = 0.002), Sk-Mel-2 (re = 52.02%, SD = 6.9%, *p* < 0.001), WM1366 (re = 98.64%, SD = 1.1%, *p* = 0.009), VMM39 (re = 79.28%, SD = 4.4% *p* < 0.001), MM485 (re = 41.41%, SD = 1.8%, *p* < 0.001), Hs940T (re = 47.59%, SD = 5.6%, *p* < 0.001), Hs852T (re = 89.34%, SD = 2.1%, *p* < 0.001), AV5 (re = 85.14% SD = 1%, *p* < 0.001), Sk-Mel-28 (re = 46.11%, SD = 3.5%, *p* < 0.001) and WM3060 (re = 92.94%, SD = 1.1%, *p* = 0.003) melanoma cell lines. (**B**) MALAT1-ASO treatment does not significantly reduce cell growth (re = 0.72%, SD = 12.4%, *p* = 0.46) in pooled primary derived human melanocytes (PHM). (**C**) MALAT1-ASO treatment significantly reduces cell growth in the MEKi-treatment resistant cell lines D04RM (re = 80.34%, SD = 2.1%, *p* = 0.01), MM415RM (re = 79.68%, SD = 1.6%, *p* = 0.001) WM3629RM (re = 89.05%, SD = 2%, *p* < 0.001) and Sk-Mel-2RM (re = 70.32%, SD = 4%, *p* = 0.003). (**D**) MALAT1 expression is not significantly altered (1.09-fold decrease, SD = 0.1, *p* = 0.29) in the MEKi-treatment resistant cell line D04RM, which was constantly exposed to 5 nM of trametinib. CT-values were normalized to GAPDH, and fold enrichment was calculated using the 2^–ΔΔCt^ method. (**E**) MALAT1-ASO treatment significantly (48-fold decrease, SD = 0.02, *p* = 0.003) reduced the ability of D04 cells to form colonies. (**F**) Representative images of 6cm-dishes of D04 cells that either received Control- or MALAT1-ASO treatment. Cells were treated for 5 days (A, B, C) or 7 days (E, F) with 50 nM final concentration of MALAT1-ASO or Control-ASO. Error bars represent standard deviation. All experiments were performed in triplicates (*n* = 3/group). Significance is shown as *p* -values calculated by Students *t*-test. ^*^ *p* < 0.05, ^**^ *p* < 0.01, ^***^ *p* < 0.001.

To determine if stable MALAT1 RNA levels are a common requirement in melanoma, we applied MALAT1-ASO treatment to a group of NRAS- and BRAF-mutant melanoma cell lines *in vitro*, including primary derived cell lines and cell lines with acquired resistance to the MEKi trametinib. First, we tested MALAT1-ASO treatment in a panel of twelve non-resistant melanoma cell lines, that have a variety of MAPK cancer-driving mutations (NRAS^Q61L^, NRAS^Q61R^, NRAS^Q61K^, NRAS^G12D^, NRAS^G12V^, BRAF^V600E^ and BRAF^D594G^), including two primary patient-derived cell lines (AV5 and Hs852T). MALAT1-ASO treatment significantly reduced cell growth in all cell lines (*p*-values ranged from <0.001 to 0.03) when compared to treatment with the non-targeting Control-ASO. (Figure 5A) Additionally, we tested MALAT1-ASO treatment on non-cancerous, patient-derived pooled human melanocytes (PHM). Notably, there were no significant cell growth-decreasing effects observed for MALAT1-ASO treatment in human melanocytes. (Figure 5B) Resistance to treatment is the main limiting factor in melanoma therapy and melanoma patients often develop resistance to small molecule kinase inhibitors such as trametinib [53]. Our next step was to explore whether melanoma cells with acquired trametinib-resistance keep their vulnerability to MALAT1-ASO treatment. We tested MALAT1-ASO treatment on four trametinib-resistant melanoma cell lines: D04RM, MM415RM, WM3629-RM and Sk-Mel-2RM. MALAT1-ASO treatment significantly reduced cell growth in all four cell lines to a similar extent as in their non-resistant, treatment naïve, counterparts. (Figure 5A, 5C) As shown earlier, short-term MEKi-therapy strongly induced MALAT1-expression in D04. (Figure 2D) The D04RM cell line acquired resistance to MEK inhibition through chronic exposure to trametinib. Previously, it was shown by analysis of phosphorylated ERK levels using immunoblot that trametinib initially reduces MAPK signaling in D04 cells, but this recovers over time [54]. To investigate potential differences in MALAT1 expression between D04RM cells and their treatment naïve counterparts, we used RT-qPCR and contrary to short term MEKi treatment, we found no significant changes in MALAT1 expression. (Figure 2E, 5D) We then aimed to identify the role of MALAT1 in the clonogenic potential of NRAS-mutant melanoma cells using a colony formation assay in the D04 cell line. Treatment with MALAT1-ASO significantly reduced colony formation, with an average of 1 colony formed, while control-ASO treatment led to an average of 48 colonies (Figure 5E, 5F).

These results suggest that inhibiting MALAT1 effectively reduces the ability of melanoma cell lines to proliferate and survive *in vitro*. The data also indicate that chronic exposure to MEKi treatment, which is accompanied by restoring of MAPK-signaling, also restores MALAT1 RNA levels. However, MEKi-resistance did not impair the cells’ vulnerability to MALAT1-ASO treatment. Our findings indicate that MALAT1 inhibition strongly reduces colony growth abilities of individual melanoma cells. Additionally, we show that healthy human melanocytes were not vulnerable to MALAT1-ASO treatment, indicating that cell growth inhibition due to MALAT1 degradation is a specific vulnerability of melanoma cells. Notably, the effectiveness of MALAT1-ASO treatment in inhibiting the survival and proliferation of NRAS- and BRAF-mutated melanoma cells is surprising in light of MALAT1 being strongly downregulated in patient samples of melanoma.

### MALAT1-ASO treatment reduces tumor growth in melanoma *in vivo*

**Figure 6:**
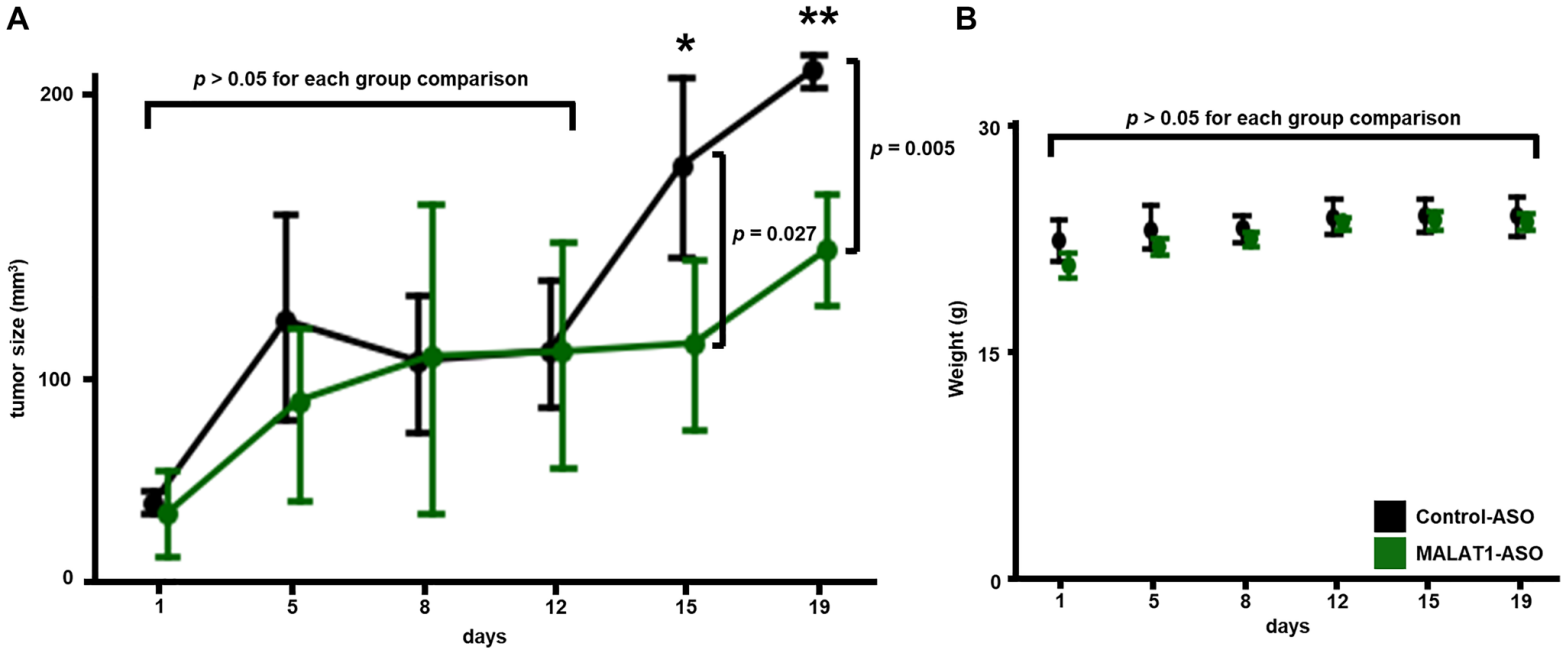
MALAT1-ASO treatment inhibits tumor growth *in vivo* and does not cause weight loss. (**A**) Mice, that were carrying subcutaneous D04 xenografts on their right flank (group size: *n* = 4), were either treated with MALAT1-ASO (green) or Control-ASO (black), twice a week, for 5 treatments. Subcutaneous ASO injections were co-applied with the transfection reagent *in vivo*-jetPEI^®^, close (~0.5 cm) to the tumor site. Significant smaller average tumor size was observed in the group of MALAT1-ASO treated mice on day 15 (MALAT1-ASO: 113 mm^3^, SD = 42 mm^3^; Control-ASO: 174 mm^3^, SD = 38 mm^3^; *p* = 0.027) and day 19 (MALAT1-ASO: 145 mm^3^, SD = 28 mm^3^; Control-ASO: 208 mm^3^, SD = 7 mm^3^; *p* = 0.005). (**B**) No significant difference in weight change in comparison of MALAT1-ASO and Control-ASO treatment groups could be observed during the treatment period. Data points represent average tumor size in (A) and average animal weight in (B). Error bars represent standard deviation. Significance is shown as *p*-values calculated by Students *t*-test. ^*^ *p* < 0.05, ^**^ *p* < 0.01, ^***^ *p* < 0.001.

To our knowledge, there is currently no data on the testing of ASO-mediated MALAT1 inhibition *in vivo* in melanoma. MALAT1-targeting ASO nanostructures have shown promising potential for the treatment of cancer metastasis *in vivo* [29]. To further investigate the anti-cancer effects of MALAT1 inhibition in MALAT1-downregulated cancer, we applied systemic MALAT1-ASO treatment in xenograft mouse models with NRAS-mutant melanoma tumors. The xenograft model was established in J:NU immunodeficient nude mice, a standard mouse model for *in vivo* drug efficacy testing. To generate xenograft tumors, 4–6-week-old mice were subcutaneously injected in the right flank with 2 × 10^6^ D04 melanoma cells embedded in matrigel. Two weeks after the establishment of the xenograft model, the mice were randomly divided in two treatment groups and treatment began with subcutaneous injections of MALAT1-ASO or non-targeting Control-ASO (150 μg ASO/mouse/treatment). The cellular uptake can be a challenge and limitation for systemic ASO delivery [55]. Therefore, ASOs were co-applied using the commercially available lipofectant reagent *in vivo*-jetPEI^®^ (60 ul/ml). The mice received two treatments per week for 15 days (5 treatments total). Before and during the treatment period, the tumor size and weight of the mice were measured twice a week. On experimental day 15, when the mice received their final treatment, the average size of tumors in the MALAT1-treatment group was significantly smaller (−35%) compared to the control (*p* = 0.03, MALAT1-ASO: 113 mm^3^, SD = 42 mm^3^ vs. Control-ASO: 174 mm^3^, SD = 38 mm^3^). Mice were sacrificed at the planned endpoint (day 19) of the experiment, when the average tumor volume in the treatment group remained significantly smaller (−30 %) compared to the control (*p* = 0.005, MALAT1-ASO: 145 mm^3^, SD = 28 mm^3^ vs. Control-ASO: 208 mm^3^, SD = 7 mm^3^). (Figure 6A) To further analyze the significance of tumor volume differences between the two xenograft groups, we additionally performed a multiple comparison correction. The calculation for Bonferroni Correction (α = 0.05, Adjusted α: 0.00833, *n* = 6) showed that after multiple comparison correction, the tumor size differences between the two groups were significant at the endpoint (day 19) of the *in vivo* experiment. During the entire period, there were no significant differences between the average weights of the two groups of mice. (Figure 6B) The above data suggests that co-application of the transfection reagent *in vivo*-jetPEI^®^ and MALAT1-ASO shows promising results in inhibiting NRAS-mutant melanoma tumor growth *in vivo*. The mice did not lose weight and tolerated the treatment well, indicating a low toxicity profile for MALAT1-ASO treatment *in vivo*. This data provides additional evidence that MALAT1 inhibition causes tumor growth reduction in cancer types that present MALAT1 downregulation.

## DISCUSSION

In this study we investigated the connections and correlations of MALAT1, and MAPK-pathway associated gene expression. Using an ASO-based approach to downregulate MALAT1, we show that MALAT1-ASO treatment specifically targets MALAT1 isoforms, with a low likelihood of causing unwanted “off-target” effects. Our findings indicate that MALAT1 may regulate RNA levels and protein expression of the gene coding for the essential MAPK signaling BRAF kinase. This is particularly of interest because BRAF is an important target for FDA-approved small molecule therapy in melanoma [44]. While further investigations will be needed to determine the mechanisms underlying BRAF downregulation upon MALAT1-inhibition, one possible explanation is that MALAT1 may regulate the transcriptional activity of BRAF through interactions with specific gene-regulatory elements, which are not present in the other MAPK genes. For instance, MALAT1 has been shown to interact with chromatin remodeling structures and to localize to a multitude of genomic sites of active genes [56, 57]. It was reported that MALAT1 regulates alternative splicing through the phosphorylation of splicing factors [58]. Therefore, another possibility for reduced BRAF mRNA and protein levels upon MALAT1-inhibition is that MALAT1 could play a regulatory role in alternative splicing of BRAF mRNA. Additionally, there is also the possibility of other unknown factors that contribute to the specificity of MALAT1 regulation of BRAF expression. Inhibition of MAPK signaling using a MEK inhibitor upregulated MALAT1 expression, while acquired resistance to MEKi not only restored MAPK-signaling but also restored MALAT1 expression. Additionally, we used a large panel of patient-derived melanoma and healthy skin tissue to present gene expression correlation of MALAT1 and genes associated with MAPK signaling. Most of the correlations were stronger in melanoma when compared to healthy skin, indicating that MALAT1 may play a role in the dysregulation of MAPK signaling in melanoma. MALAT1 expression significantly correlated with NRAS expression. However, MALAT1-inhibition did not significantly affect NRAS RNA levels in NRAS-mutant melanoma cells. One possible explanation for this observation is that the observed correlation could reflect a more complex interplay between MALAT1 and NRAS expression in the tumor microenvironment, which cannot be recapitulated *in vitro* using cell lines. Another possibility is that there may be other factors, such as other non-coding RNAs or signaling pathways, that regulate NRAS expression and compensate for the loss of MALAT1 in melanoma cell lines. Based on these findings, we propose the hypothesis that MALAT1 may have differential effects on the expression levels of genes in the MAPK pathway. Specifically, while MALAT1 may directly regulate the transcription of BRAF, its regulatory interplay with NRAS, MEK1/2, and ERK1/2 may be mediated through indirect mechanisms, such as shared upstream regulatory dependencies. Further investigation is necessary to fully elucidate the nature of these regulatory interactions.

MALAT1-ASO treatment strongly impaired cell growth and colony formation in a large panel of NRAS- and BRAF-mutated melanoma cells, including MEKi-resistant cells. We present evidence that treatment resistance to MAPK-inhibiting small molecule therapy (MEKi) does not affect vulnerability to MALAT1-ASO treatment *in vitro*. Furthermore, we showed that MALAT1-ASO treatment significantly impaired tumor growth *in vivo* in a xenograft NRAS-mutant melanoma mouse model. *In vivo* ASO treatment was applied with the transfection reagent *in vivo*-jetPEI^®^, which is currently trials as a transfection reagent for undergoing clinical immunomodulatory therapy (IFx-Hu2.0) for advanced melanoma, Merkel cell carcinoma, and advanced cutaneous squamous cell carcinoma (https://clinicaltrials.gov/ identifier: NCT04853602). No obvious signs of toxicity could be measured *in vivo*, indicating that MALAT1-ASO treatment has a low toxicity profile and that the dosage that was used in this study could potentially even be increased.

Previous studies have identified the role of MALAT1 in the transcriptional and post-transcriptional regulation of oncogene expression [59]. It has been reported that MALAT1 can support either oncogenesis through gene expression upregulation or tumor suppression through gene expression downregulation. These findings have been highly divergent across different cancer types, leading to the hypothesis that MALAT1-dependent gene regulation relies on its distinct interaction partners, which can differ greatly among different cancer types [60]. However, little is known about the potential tissue-specific role of MALAT1 in melanoma. In this study, we present a novel “paradigm of MALAT1”. In addition to showing that ASO-mediated MALAT1 degradation significantly inhibits melanoma cell survival, we also observed strong downregulation of MALAT1 in melanoma when compared to healthy skin. Moreover, MALAT1 expression also was significantly decreased in melanoma compared to healthy skin when put in relationship to gene expression of the MAPK pathway genes NRAS, BRAF, MEK1, MEK2, ERK1, and ERK2. Expanding the analysis to other cancer types shows that MALAT1 expression is also downregulated in hepatocellular carcinoma and squamous cell carcinoma of the lung. All these cancer types share two main features: (i) MALAT1 was reported to have direct regulatory functions on the MAPK pathway and (ii) MALAT1 inhibition caused disadvantageous reactions in the respective cancer cells. This patient sample analysis is also in accordance with our finding that drug-induced MAPK-inhibition causes dose-dependent MALAT1-upregulation in NRAS-mutant melanoma. Patient-derived datasets of melanoma, hepatocellular carcinoma, non-small cell lung cancer, and their respective adjacent healthy tissue controls have been previously used to show cancer-specific MALAT1-upregulation [12, 52, 61–65]. However, to our knowledge, ours is the first study to utilize large-scale TCGA and GTEx datasets to compare MALAT1-expression in these types of cancer and show MALAT1 downregulation in cancerous tissue compared to healthy tissue. Previously presented cancer-specific MALAT1-upregulation in melanoma was executed by comparing biopsies of close distance derived from the same patients (paired samples). We hypothesize that these results rather reflect expressional changes in the tumor microenvironment and its directly adjacent tissue. Such changes may include MALAT1 downregulation in healthy tissue rather than upregulation in cancer. Meanwhile, our approach incorporates a global perspective on MALAT1 expression in both patient-derived cancerous and healthy skin samples from large distinct cohorts of tissue donors.

The behavior of oncogenes in carcinogenesis and cancer complexity must be placed within a global context and cannot be described by linear connections regarding up- and downregulation of one single gene. Decades of cancer research have unveiled anomalies and paradigms that help us better understand the mechanisms underlying this disease. Oncologic paradigms can open new ways to explore and understand the development and homeostasis of cancer cells. As Bizzarri et al. aptly stated, we must recognize that “a thorough and constant transformation of our scientific *weltanschauung* is urgently needed” [66]. Thus, we unveil a novel paradigm of MALAT1 expression. Oncogenic functions of certain cancer types seem to be dependent on MALAT1 expression, but not necessarily on MALAT1 upregulation. MALAT1 seems to be downregulated in melanoma, while simultaneously exercising essential oncogenic functions. RAS/RAF/MEK/ERK-pathway upregulation is the main mechanism of survival in melanoma and successful melanoma therapy strongly focuses on inhibition of members of this MAPK-signaling pathway. Although the precise role of MALAT1 in MAPK-signaling remains largely unknown, our findings suggest the presence of multiplexed oncogenic functions of MALAT1 in melanoma and other types of cancer.

## MATERIALS AND METHODS

### Cell culture

The human melanoma cell line VMM39 (NRAS-Mutation: Q61L, Q61R) was purchased from American Type Culture Collection (ATCC^®^). The human melanoma cell lines D04 (NRAS-Mutation: Q61L), MM415 (NRAS-Mutation: Q61L), WM1366 (NRAS-Mutation: Q61L), WM3629 (BRAF-Mutation D594G, NRAS-Mutation: G12D), Sk-Mel-2 (NRAS-Mutation: Q61R), WM3060 (NRAS-Mutation: Q61K) and Sk-Mel-28 (BRAF-Mutation V600E) were gifted by Dr. Boris Bastian at the UCSF. The primary human melanoma cell lines Hs940T (NRAS-Mutation: Q61R) and Hs852T (NRAS-Mutation: G12V) were purchased from the Cell and Genome Engineering Core (CGEC) at the UCSF. The primary human melanoma cell-line AV5 (BRAF-Mutation: V600E) was obtained from metastasis of a melanoma patient. All experimental protocols were approved by UCSF Human Research Protection Program Institutional Review Board (IRB# 12-0948). Methods were carried out in accordance with relevant guidelines and regulations. The resistant cell lines D04RM, MM415RM, Sk-Mel-2RM and WM3629RM were established as previously described [54]. The primary human melanocytic cell line (PHM) from infant foreskin of five healthy donors was available in our cell repository. Melanoma cell-lines were maintained in RPMI 1640 media supplemented with 10% (vol/vol) heat-inactivated fetal bovine serum. Melanocytes were maintained in M254 medium with HMGS supplements (1x final solution). All cell lines were incubated at 37°C under 5% CO_2_.

### RNA secondary structure

RNA secondary structures and minimum free energy (MFE) structures were obtained using the RNAfold Web Server (University of Vienna, http://rna.tbi.univie.ac.at/cgi-bin/RNAWebSuite/RNAfold.cgi). The MFE is predicted using a dynamic programming algorithm described by Zuker et al., [67].

### RNA extraction and quantitative real-time PCR (RT-qPCR)

TRIzol^™^ Solution (Thermo Fisher Scientific^®^) was used for extracting Total RNA from cells and tissues according to the manufacturer’s instructions. Total RNA was quantified by NanoDrop^™^ ND-1000 (Thermo Fisher Scientific^®^) or Quibit^™^ 4 (Thermo Fisher Scientific^®^). 50 ng or RNA was reverse transcribed using the cDNA synthesis and gDNA removal QuantiTect^®^ Reverse Transcription Kit (Thermo Fisher Scientific^®^). Real time PCR was performed using the iTaq^™^ Universal SYBR^®^ Green Supermix (Bio-Rad Laboratories, Inc.), 10 ng of cDNA and on a QuantStudio^™^ 5 Real-Time PCR System or a 7500 fast real time PCR system (both from Thermo Fisher Scientific^®^). Relative gene expression was calculated using the comparative Ct method, normalized to GAPDH. Primers are listed in Supplementary Table 3. Primers were obtained from Integrated DNA Technologies, Inc.

### Oligonucleotide transfection

ASO-GapmeRs were purchased from QIAGEN N.V. The following ASO-sequences (5′-3′) were used: MALAT1-targeting ASO sequence TAAAGCCTAGTTAACG and the non-targeting control sequence AACACGTCTATACGC (QIAGEN N.V. standard) were used. Final concentrations of ASOs in media are listed in figure legends and for *in vitro* experiments, the transfection reagent Lipofectamine^™^ 3000 (2 ul/ml) was added according to the manufacturer’s instructions.

### Expression analysis in TCGA and GTEx

The analysis of TCGA/GTEx gene expression data was done in R. For TCGA data, the following datasets were used: SKCM dataset, filtered for NRAS- and BRAF-mutated melanoma (*n* = 366), LIHC dataset (*n* = 424), LUAD dataset (*n* = 585) and LUSC dataset (*n* = 550) was used. The GDCquery function of the TCGAbiolinks package was run with the following parameters: project = “TCGA-SKCM”, data.category = “Transcriptome Profiling”, data.type = “Gene Expression Quantification”, workflow.type = “HTSeq – FPKM”. GDCdownload and GDCprepare then produce a RangedSummarizedExperiment. Expression values are then stored in a data frame and converted to TPM by dividing each FPKM-value by the total FPKM of each sample and multiplying by 10^6^. To retrieve GTEx data for skin (*n* = 1305), liver (*n* = 226) and lung (*n* = 578)“GTEx_Analysis_2017-06-05_v8_RNASeQCv1.1.9_gene_tpm.gct.gz” was downloaded from gtexportal.org/home/datasets.65 Skin samples within the GTEx dataset were identified by referencing “GTEx_Analysis_v8_Annotations_SampleAttributesDS.txt”. For both TCGA and GTEx, duplicate genes were removed. If a patient provided multiple specimens, only the first would be used. All TPM values were normalized by dividing by the TPM of GAPDH for each sample. Cor.test was applied to find the correlation between each gene and MALAT1. Spearman’s correlation coefficient (ρ) was used to measure rank correlation.

### Cell viability assay

Dependent on cell doubling time, 0.7–2 × 10^3^ cells were seeded in 96 well-plates one day prior to transfection. One day after seeding, cells were incubated in media with the final oligonucleotide concentration and transfection reagent. Five days after transfection, total luminescence was measured on the Synergy^™^ HT (Agilent Technologies Inc.) plate reader using Promega^®^ CellTiter-Glo^®^ and Gen5 software. Cell growth is shown in relation to cells incubated with the Control-ASO.

### Colony formation assay

1 × 10^3^ cells were seeded in six well-plates. One day after seeding, cells were incubated in media with 50 nM oligonucleotide concentration and transfection reagent. Six days after transfection, cells were washed with PBS, fixed with 10% neutral buffered formalin, and stained with 0.1% crystal violet solution. Colonies were defined as cell conglomerates with >50 cells. Digital images of plates were evaluated by two independent reviewers for colony counts. The final counts were calculated as the average count of both reviewers for all triplicates.

### Statistics and reproducibility

Error bars in all the plots indicate mean ± S.D. *P*-value < 0.05 was considered statistically significant. ^***^ *p*-value < 0.001, ^**^ *p*-value < 0.01, ^*^ *p*-value < 0.05 by one tailed, paired Student’s *t*-test. ΔΔCt values were used to calculate *p*-values for gene expression comparison. All experiments were performed at least three times, unless otherwise indicated. Statistics was calculated with Microsoft^®^ Excel Version 2107.

### Animal models

Rodent experimental procedures were approved by the Office of Research Institutional Animal Care and Use Program (IACUC, Chair: Jeremy Lieberman, MD, USA) at the University of San Francisco (UCSF). All *in vivo* studies were conducted under the authorized protocol number AN174613-03. Mice were maintained in a pathogen-free environment and had free access to food and water. 2 × 10^6^ D04 (*n* = 3/group) cells in 150 μl of PBS and 50 μl of Matrigel were subcutaneously injected on the right posterior dorsal flank of 4- to 6-week-old homozygous nude Foxn1^nu^/Foxn1^nu^ mice (Stock.no 007850, J:NU). Mice were obtained from JAX^®^. Tumor size was measured using a digital caliper and the formula 0.5 × (length × (width^2)) was used to calculate tumor volume. Mice were treated twice a week with 150 μg of MALAT1-ASOs, or non-targeting control ASOs and 9.6 μl of *in vivo* JetPEI^®^ diluted in an overall amount of 200 μl of 5% glucose. ASO injections were applied subcutaneously, close to the tumor site (~5 mm), but not intratumorally. Mice were weighed twice a week and observed for signs of distress or disorder. All experiments were performed in accordance with the UCSF Laboratory Animal Resource Center (LARC) guidelines.

### Protein extraction and immunoblotting

1 × 10^5^ D04 cells were seeded in six well-plates one day prior to transfection. Cells were incubated in media with a final oligonucleotide concentration of 50 nM. Total protein lysates were homogenized in 1x RIPA buffer, and Halt protease and phosphatase inhibitor cocktail (Thermo Fisher Scientific^®^) followed by centrifugation at 14,000 RPM/minute at 4°C. Protein concentration was quantified using the Pierce^™^ BCA Assay Kit (ThermoFisher Scientific^®^). Linear absorbance was measured using the Synergy^™^ HT (Agilent Technologies Inc.) plate reader. Total protein in 1 × Laemmli buffer with 10% 2- mercaptoethanol was separated by SDS/PAGE, transferred for 15 h to a PVDF membrane (IPVH00010; MilliporeSigma^®^) by electroblotting with 20% (vol/vol) methanol, and blocked for 1 h in in Intercept (TBS) blocking buffer (LI-COR^®^). Membranes were incubated for two days at 4°C with primary antiserum for BRAF (Cell Signaling Technology^®^, cat.no.: 9433, dilution 1:1000) and β-Actin (Cell Signaling Technology^®^, cat.no.: 8457, dilution 1:2500) following incubation with secondary Goat Anti-Rabbit and Anti-mouse serum (LI-COR^®^, dilution 1:5000) for 1 h and scanned using the Li-COR^®^ Odyssey^®^ Imaging system.

## SUPPLEMENTARY MATERIALS



## Abbreviations

lncRNA: Long Non-coding RNA
ASO: Antisense Oligonucleotide
NEAT2: Non-coding Nuclear-enriched Abundant Transcript 2
MALAT1: Metastasis Associated Lung Adenocarcinoma Transcript 1
siRNA: Small Interfering RNA
RTK: Receptor Tyrosine Kinase
FDA: U.S. Food and Drug Administration
GTEx: The Genotype-Tissue Expression project
TCGA: The Cancer Genome Atlas
LIHC: Liver Hepatocellular Carcinoma
LUAD: Lung Adenocarcinoma
LUSC: lung squamous cell carcinoma
MEKi: MEK-inhibitor
