# Supplementary material for: Deconstructing the role of MALAT1 in MAPK-signaling in melanoma: insights from antisense oligonucleotide treatment
Source: Oncotarget. 2023 May 26;14:543–60. doi: 10.18632/oncotarget.28447 (PMC10219656; doi:10.18632/oncotarget.28447)
Supplement: Supplementary file 1 [file oncotarget-14-28447-s001.pdf]

Deconstructing the role of MALAT1 in MAPK-signaling in melanoma: insights from antisense oligonucleotide treatment

SUPPLEMENTARY MATERIALS

Supplementary Table 1: MALAT1 isoforms have low coding potential

MALAT1 Isoforms	length (bp)	Protein coding potential metrics							
		PRIDE reprocessing 2.0		PhyloCSF score		CPAT coding probability		Bazzini small ORFs	
		Raw result	Interpretation	Raw result	Interpretation	Raw result	Interpretation	Raw result	Interpretation
ENST00000534336	8762	0	non-coding	−38.4593	non-coding	2.56%	non-coding	1	non-coding
ENST00000619449	5340	0	non-coding	−54.9455	non-coding	1.84%	non-coding	0	non-coding
ENST00000508832	1519	0	non-coding	−28.0879	non-coding	0.45%	non-coding	0	non-coding
ENST00000618132	725	0	non-coding	−40.8265	non-coding	0.08%	non-coding	0	non-coding
ENST00000618227	593	0	non-coding	−74.1569	non-coding	0.41%	non-coding	0	non-coding
ENST00000616691	587	0	non-coding	−84.4973	non-coding	6.15%	non-coding	0	non-coding
ENST00000610851	584	0	non-coding	−74.1569	non-coding	1.27%	non-coding	0	non-coding
ENST00000616527	572	0	non-coding	−108.3966	non-coding	9.52%	non-coding	1	non-coding
ENST00000544868	480	0	non-coding	−606.7062	non-coding	1.45%	non-coding	1	non-coding
ENST00000618925	424	0	non-coding	−52.3196	non-coding	0.39%	non-coding	0	non-coding
ENST00000617791	394	0	non-coding	−366.659	non-coding	5.77%	non-coding	0	non-coding
ENST00000610481	353	0	non-coding	–	non-coding	3.62%	non-coding	0	non-coding
ENST00000620902	352	0	non-coding	–	non-coding	0.93%	non-coding	0	non-coding
ENST00000620465	333	0	non-coding	−99.0741	non-coding	0.55%	non-coding	0	non-coding
ENST00000617489	318	0	non-coding	−84.4973	non-coding	6.23%	non-coding	0	non-coding
ENST00000612781	234	0	non-coding	−99.0741	non-coding	0.03%	non-coding	0	non-coding
ENST00000613376	132	N/A	N/A	N/A	N/A	N/A	N/A	N/A	N/A

Listed are the 17 MALAT1 isoforms and their length as annotated in the ensemble gene-annotation GRCH38.p13 (human) and the LNCIPEDIA-database (V 5.2). Conservation and coding probability scores for MALAT1-isoforms, calculated by the four different bioinformatic toolsets PRIDE reprocessing 2.0, PhyloCSF, CPAT nd Bazzini small ORFs. Positional locus conservation is defined by the flanking protein coding genes and their orthology compared to the human lncRNA, assessed by the Emsembl Compara API.

Supplementary Table 2: Comparison of the MALAT1-ASO sequence TAAAGCCTAGTTAACG to its top 15 hits in the human transcriptome shows high on-target affinity to three MALAT1 isoforms and low off-target binding affinity to other RNA-transcripts

Rank	Transcript name	species	Accession Nr	RNA type	Total score	Query cover	E value	miss matches
**1**	MALAT1-1	Homo Sapiens	NR_002819.4	non protein-coding	32.2	100%	1.5	0
**2**	MALAT1-2	Homo Sapiens	NR_144567.1	non protein-coding	32.2	100%	1.5	0
**3**	MALAT1-3	Homo Sapiens	NR_144568.1	non protein-coding	32.2	100%	1.5	0
**4**	MRGBP-ncRNA	Homo Sapiens	NR_136405.2	non protein-coding	46.6	81%	90	3
**5**	MRGBP-mRNA	Homo Sapiens	NM_018270.6	protein-coding	46.6	81%	90	3
**6**	PIK3CB-2	Homo Sapiens	NM_001256045.2	protein-coding	24.3	75%	354	4
**7**	HAUS2-2	Homo Sapiens	NM_001130447.2	protein-coding	24.3	75%	354	4
**8**	ZNF283-2	Homo Sapiens	NM_001297752.2	protein-coding	24.3	75%	354	4
**9**	HAUS2-5	Homo Sapiens	NM_001323631.2	protein-coding	24.3	75%	354	4
**10**	HAUS2-4	Homo Sapiens	NM_001323630.2	protein-coding	24.3	75%	354	4
**11**	HAUS2-6	Homo Sapiens	NM_001323632.2	protein-coding	24.3	75%	354	4
**12**	CUL1-2	Homo Sapiens	NM_001370660.1	protein-coding	24.3	75%	354	4
**13**	C11orf16	Homo Sapiens	NM_020643.3	protein-coding	24.3	75%	354	4
**14**	HAUS2-1	Homo Sapiens	NM_018097.3	protein-coding	24.3	75%	354	4
**15**	ZNF283-1	Homo Sapiens	NM_181845.2	protein-coding	24.3	75%	354	4

Supplementary Table 3: List of all primers used for RT-qPCR

Target	Forward	Reverse
MALAT1	GACGGAGGTTGAGATGAAGC	ATTCGGGGCTCTGTAGTCCT
NRAS	ATGACTGAGTACAAACTGGTGGT	CATGTATTGGTCTCTCATGGCAC
BRAF	CCCCAAGTCACCACAAAAACC	CGGACTGTAACTCCACACCTT
MEK1	CAATGGCGGTGTGGTGTTC	GATTGCGGGTTTGATCTCCAG
MEK2	CCAAGGTCGGCGAACTCAAA	TCTCAAGGTGGATCAGCTTCC
ERK1	CTACACGCAGTTGCAGTACAT	CAGCAGGATCTGGATCTCCC
ERK2	TACACCAACCTCTCGTACATCG	CATGTCTGAAGCGCAGTAAGATT
GAPDH	TGGAAGGACTCATGACCACA	GCCATCACGCCACAGTTT
